# Supplementary material for: Achiral hard bananas assemble double-twist skyrmions and blue phases
Source: Nat Commun. 2024 Aug 8;15:6780. doi: 10.1038/s41467-024-50935-4 (PMC11310516; doi:10.1038/s41467-024-50935-4)
Supplement: Supplementary file 1 — Supplementary Information [file 41467_2024_50935_MOESM1_ESM.pdf]

Supplementary Information

Achiral Hard Bananas Assemble Double-Twist Skyrmions and Blue Phases

Rodolfo Subert[1], Gerardo Campos-Villalobos[1], and Marjolein Dijkstra[1,2*]

[1]*Soft Condensed Matter & Biophysics,*

Debye Institute for Nanomaterials Science, Utrecht University,

Princetonplein 5, 3584 CC Utrecht, The Netherlands. and

[2] *International Institute for Sustainability with Knotted Chiral Meta Matter (SKCM2),*

Hiroshima University, 2-313 Kagamiyama,

Higashi-Hiroshima, Hiroshima, 739-8527, Japan

* m.dijkstra@uu.nl

This PDF file includes Supplementary Figures 1-4.

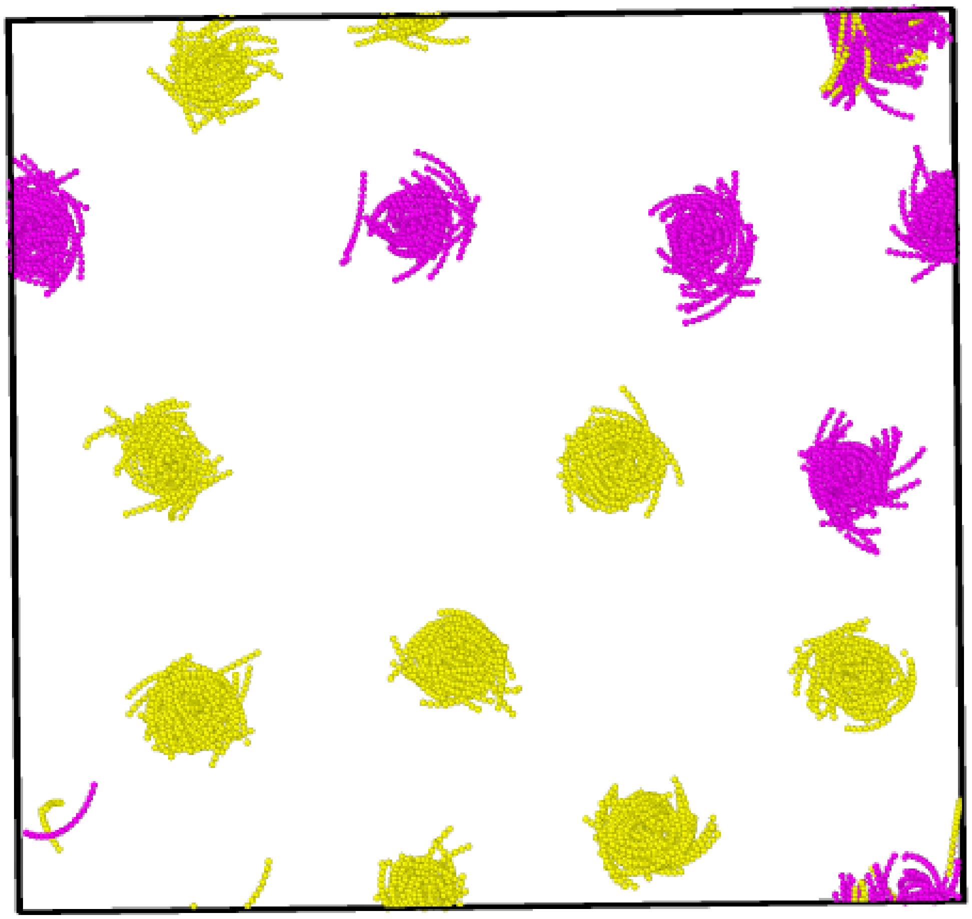

FIG. 1. **Supplementary Figure 1. Handedness of the skyrmion filaments.** The hexagonal skyrmion lattice viewed from the top. The skyrmion filaments are colored according to the sign of the twist order parameter $\mathcal{T}$, denoting the handedness of the skyrmions.

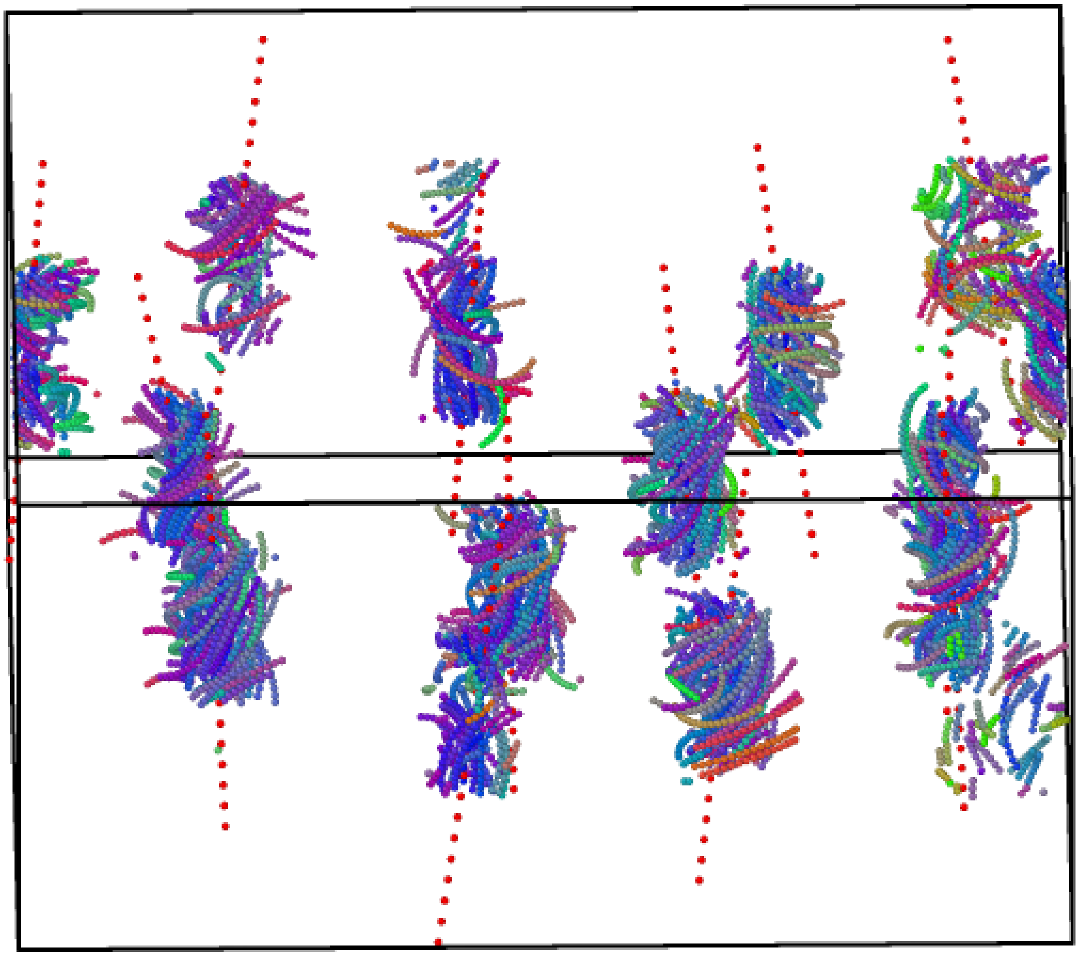

FIG. 2. **Supplementary Figure 2. Hexagonal skyrmion lattice.** A typical configuration of an hexagonal skyrmion lattice, displaying the individual skyrmion filaments. The axis of the skyrmion filaments denoted by the red spheres are determined as described in the Methods section and used as input in the calculation of the skyrmion radii.

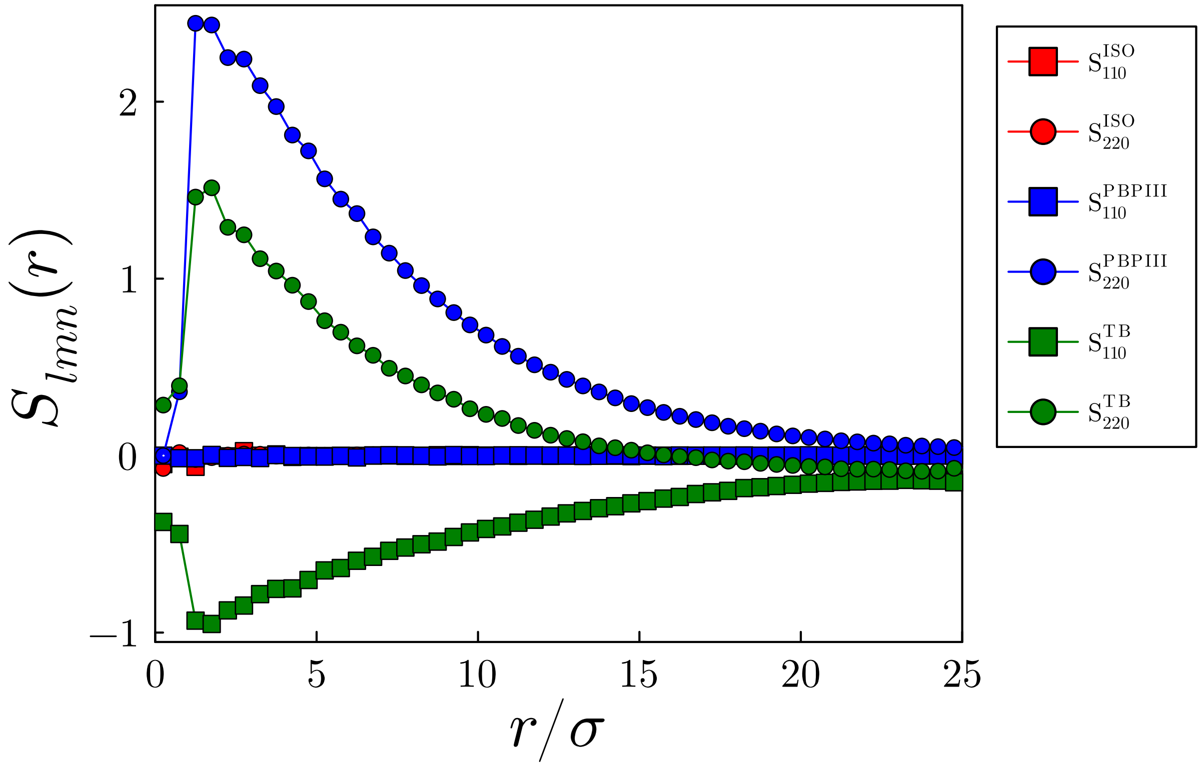

FIG. 3. **Supplementary Figure 3. Orientational correlation functions.** The odd $S_{110}(r) = -\frac{1}{\sqrt{3}}\sum_{i,j}(\hat{\boldsymbol{u}}_i \cdot \hat{\boldsymbol{u}}_j)\delta(r - r_{ij})$ and even $S_{220}(r) = \frac{3}{2\sqrt{5}}\sum_{i,j}((\hat{\boldsymbol{u}}_i \cdot \hat{\boldsymbol{u}}_j)^2 - 1)\delta(r - r_{ij})$ orientational correlation functions, with $\hat{\boldsymbol{u}}_i$ and $\hat{\boldsymbol{u}}_j$ being the banana long axis, as a function of distance r for an isotropic (ISO), polar blue phase III (PBPIII), and a twist-bend nematic (TB) phase.

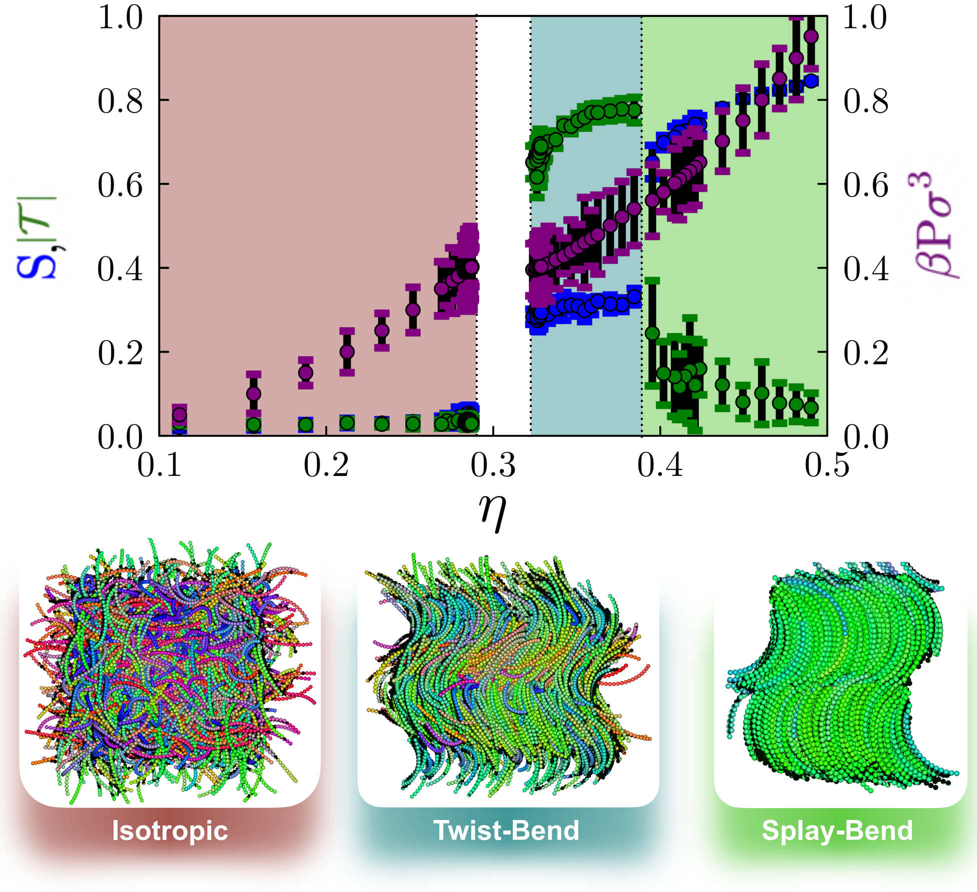

FIG. 4. **Supplementary Figure 4. Bulk phase diagram of banana-shaped particles with standard deviation.** The pressure $\beta P\sigma^3$ (purple), nematic order parameter S (blue), and heliconical order parameter $\mathcal{T}$ (green) as a function of the packing fraction η of banana-shaped particles with a length-to-diameter ratio $L/\sigma = 16$ and opening angle $\psi = 1.6$ as obtained from MD simulations. The phase diagram displays an isotropic (I) phase at low density, a twist-bend nematic ($\mathrm{N_{TB}}$) phase at intermediate density, and a splay-bend smectic ($\mathrm{Sm_{SB}}$) phase at high density. Typical configurations are shown in the panels below, where the particles are color-coded according to their orientation. The standard deviation is always at least at the second decimal, except a few measurement of the twist order parameter in the splay-bend phase.
