# Supplementary material for: Achiral hard bananas assemble double-twist skyrmions and blue phases
Source: Nat Commun. 2024 Aug 8;15:6780. doi: 10.1038/s41467-024-50935-4 (PMC11310516; doi:10.1038/s41467-024-50935-4)
Supplement: Supplementary file 3 — Description of Additional Supplementary Files [file 41467_2024_50935_MOESM3_ESM.pdf]

Description of Additional Supplementary Files

Achiral Hard Bananas Assemble Double-Twist Skyrmions and Blue Phases

Rodolfo Subert[1], Gerardo Campos-Villalobos[1], and Marjolein Dijkstra[1,2*]

[1] *Soft Condensed Matter & Biophysics,*

Debye Institute for Nanomaterials Science, Utrecht University,

Princetonplein 5, 3584 CC Utrecht, The Netherlands. and

[2] *International Institute for Sustainability with Knotted Chiral Meta Matter (SKCM2),*

Hiroshima University, 2-313 Kagamiyama,

Higashi-Hiroshima, Hiroshima, 739-8527, Japan

* m.dijkstra@uu.nl

This PDF file includes Descriptions of Supplementary Movies 1-4.

a. File Name: Supplementary Movie 1

Description:

The movie shows the nucleation of an hexagonal skyrmion lattice starting from an isotropic configuration over a time interval of $3 \times 10^5 \tau_{\mathrm{MD}}$.

b. File Name: Supplementary Movie 2

Description:

The movie shows the diffusion of the banana-shaped particles in a skyrmion lattice. To improve the visualization we only show the core of each skyrmion. Furthermore, we color all the bananas yellow in one of the skyrmion filaments in the first frame, keeping track of those bananas throughout the remainder of the movie. The movie spans a time interval of $3 \times 10^5 \tau_{\mathrm{MD}}$.

c. File Name: Supplementary Movie 3

Description:

The movie shows the effect of a square wave oscillating electric field on a skyrmion phase. As soon as the field is activated the system switches to a uniaxial nematic phase. Next, as the field is switched off, skyrmions are nucleated again.

d. File Name: Supplementary Movie 4

Description:

The movie shows an instance of PBPIII with fluctuating skyrmions after compression from an isotropic phase to a packing fraction $\eta = 0.311(2)$. The system is replicated one time along the x and y axis to better show the full length of the skyrmions through the periodic boundary conditions and spans a time interval of $3 \times 10^5 \tau_{\mathrm{MD}}$.
